# Cardiovascular flukes (Trematoda: Spirorchiidae) in *Caretta caretta* Linnaeus, 1758 from the Mediterranean Sea

**DOI:** 10.1186/s13071-017-2396-x

**Published:** 2017-10-10

**Authors:** Erica Marchiori, Enrico Negrisolo, Rudi Cassini, Luisa Garofalo, Lisa Poppi, Cinzia Tessarin, Federica Marcer

**Affiliations:** 1 Department of Animal Medicine, Production and Health, University of Padova, Viale dell’Università, Legnaro, Italy; 2 Department of Comparative Biomedicine and Food Science, University of Padova, Viale dell’Università, Legnaro, Italy; 3 Istituto Zooprofilattico Sperimentale delle Regioni Lazio e Toscana, Centro di Referenza Nazionale per la Medicina Forense Veterinaria, Rieti, Italy

**Keywords:** Sea turtles, *Caretta caretta*, Cardiovascular flukes, Spirorchiidae, Mediterranean Sea, Phylogeny

## Abstract

**Background:**

The northern Adriatic Sea represents one of the most important neritic foraging grounds for the loggerhead sea turtle *Caretta caretta* L. in the Mediterranean Sea. Four genera of blood flukes with variable prevalence and pathogenic impact have been reported worldwide in this species. *Hapalotrema* Looss, 1899 and *Amphiorchis* Price, 1934 are the only two genera reported in Mediterranean waters; however, updated data describing spirorchiidiasis in the central and eastern Mediterranean and infection prevalence are still lacking. This work aimed to investigate the presence and pathology of spirorchiidiasis in *C. caretta* in the Mediterranean Sea.

**Methods:**

One hundred sixty-eight animals stranded along the northwestern Adriatic coast between 2009 and 2015 were submitted to necropsy and subsequent analyses for the detection of adult flukes, detection of eggs in the faeces and spleen and histopathology. Molecular analyses were carried out on hosts (mitochondrial D-loop) and parasites (28S gene and ITS2 spacer) to trace the turtle origins and identify the fluke phylogenetic relationships.

**Results:**

Spirorchiidiasis was detected in 16.7% of the animals. *Hapalotrema mistroides* (Monticelli, 1899) and *Neospirorchis* sp. were found in twenty-six and ten cases, respectively. Adult flukes were found in six cases, while eggs were detectable through copromicroscopic examination for all infected turtles, and the results for the detection of eggs in the spleen agreed with the copromicroscopic analysis. Only mild lesions were observed. Eggs of types 1 and 3 were grossly visible in the gastrointestinal mucosa, vasculitis was rarely observed in the heart and great vessels, and multifocal granulomas were widespread in the tissues. Molecular identification unambiguously assigned the spirorchiid samples to *H. mistroides* and *Neospirorchis* sp. Genetic characterization of loggerhead mtDNA pointed to a Mediterranean origin of the turtle hosts.

**Conclusion:**

This survey provides new data on the spread of spirorchiidiasis in the Mediterranean loggerhead sea turtle population and reports for the first time the presence of *Neospirorchis* spp. in this basin. The infections did not have a causal effect on the death nor a strong impact on the general health status of the animals.

**Electronic supplementary material:**

The online version of this article (10.1186/s13071-017-2396-x) contains supplementary material, which is available to authorized users.

## Background

Infection by blood flukes of the family Spirorchiidae Stunkard, 1921 causes morbidity and mortality in marine turtle populations worldwide. The presence of adult flukes in the circulatory system of infected animals and the embolization of eggs through host vessels leads to lesions of varying severity, including arteritis, thrombosis, aneurysms of the great vessels and disseminated granulomas in all body districts [1–3].

Six genera of spirorchiids are currently recognized as parasites of marine turtles worldwide: *Hapalotrema* Looss, 1899; *Neospirorchis* Price, 1934; *Carettacola* Manter & Larson, 1950; *Amphiorchis* Price, 1934; *Learedius* Price, 1934; and *Monticellius* Mehra, 1939. Most of the reports of spirorchiidiasis in the marine environment concern the green turtle (*Chelonia mydas* Linnaeus, 1758); however, the infection is also well documented in the loggerhead sea turtle (*Caretta caretta* Linnaeus, 1758) in which species of the genera *Hapalotrema*, *Neospirorchis*, *Carettacola* and *Amphiorchis* have been reported.

Studies on the prevalence of infection in *C. mydas* report high percentages of positivity worldwide, ranging from 80 to 100% from Australia to Florida (USA) [3–9]. Similar surveys carried out on *C. caretta* from the north-western Atlantic region describe the prevalence as ranging between 33 and 96% [1, 4].

The genus *Hapalotrema* is globally distributed and is described in Florida and Australia [1, 4, 10–13]. Three species, i.e. *H. synorchis* Luhman, 1935, *H. pambanensis* Gupta & Mehrotra, 1981 and *H. mistroides* (Monticelli, 1989), have been reported in loggerhead sea turtles from both Florida and western Australia. Species of *Neospirorchis* have been isolated from *C. caretta* in the north-western Atlantic region [1, 11, 14, 15]. *Neospirorchis pricei* Manter & Larson, 1950 was morphologically identified by Manter & Larson [14] and by Stacy [11]; however, in the latter study molecular and phylogenetic analyses revealed the presence of other unidentified species among the collected specimens with different localization in the definitive hosts [11]. Species of the genus *Carettacola* have also been reported from the western Atlantic coast [4, 14], whereas there is only one report of *Amphiorchis* in *C. caretta* from the Mediterranean Sea [16]*.*

Few descriptions of spirorchiids in loggerhead sea turtles and green turtles exist for the Mediterranean area, and information on infection prevalence is not available. In the late nineteenth century, Monticelli described the blood fluke *Mesogonimus constrictus* (Leared, 1862) (syn. *Hapalotrema mistroides*) in a specimen of *C. caretta* (syn. *Thalassochelys caretta* Bonaparte, 1838) from the Gulf of Naples, off Italy [17], and the same species was reported by other authors in both loggerhead and green turtles from the Egyptian coast a few years later [18–21]. Recently, Santoro et al. [22] described severe lesions due to *H. mistroides* infection in a loggerhead sea turtle stranded along the Italian Tyrrhenian coastline. *Amphiorchis* spp. associated with severe disease were recently reported by Cribb et al. [16] in neonate loggerhead sea turtles kept in a facility in Valencia, Spain. No reports of the genera *Neospirorchis* and *Carettacola* are available for the Mediterranean Basin.

Methods for postmortem diagnosis include gross and microscopic observations of adult flukes, egg masses, and related lesions in the organs, tissues and vessels [2]. Other laborious techniques can be used to detect adult flukes in blood and organs [23] and to search for spirorchiid eggs in the spleen [7]; copromicroscopic examination has also been used to detect the presence of eggs in faecal material [1].

Parasite identification is achieved through observations of morphological features. However, intact adult parasites are often difficult to obtain from stranded animals, and success also depends on the preservation status of the carcasses [4]. The eggs represent the most resistant stage and have been classified into three types [1]: eggs with bipolar filaments (type 1) are attributed to different genera, i.e. *Hapalotrema*, *Learedius*, *Monticellius* and *Amphiorchis*; eggs with monopolar filament (type 2) are described for *Carettacola*; and round eggs (type 3) are attributed to *Neospirorchis* [1, 24]. Thus, egg morphology alone is a helpful but limited tool for parasite identification [4]. Molecular approaches are therefore important for overcoming the limits of morphological identification and are the basis for analyses of phylogenetic relationships within the family Spirorchiidae [11, 16, 25, 26]. Considering these factors, the aims of this work were to (i) obtain prevalence data on spirorchiidiasis in the central Mediterranean population of *C. caretta* using different diagnostic approaches; (ii) evaluate their pathogenic impacts on the host; and (iii) increase our knowledge for the identification of blood flukes by morphological and molecular methods.

## Methods

Between June 2009 and November 2015, 168 loggerhead sea turtles were found stranded and dead along the north-western Adriatic coast of Italy from Grado (Udine Province; 45°41′N, 13°24′E) to Riccione (Rimini Province; 44°00′N, 12°39′E). Necropsy and parasitological examination were performed on the carcasses at the Department of Comparative Biomedicine and Food Sciences and the Department of Animal Medicine, Productions and Health of Padova University (Italy), respectively. Permission for the execution of necropsies on stranded sea turtles was endorsed by the local health authority to the University of Padova.

### Anatomopathological analyses

Necropsies were performed following guidelines given by Flint et al. [2] and Poppi et al. [27]. Before starting the dissections, morphometric data were collected that included SCL (straight carapace length) measurements. A body condition score (BCS) was assigned to the carcasses after evaluation of the adipose tissue covering the ventral muscles. In animals that were freshly dead (*n* = 11) or in moderate decomposition (*n* = 99), all organs (adrenal gland, gastrointestinal tract, heart and major vessels, kidney, liver, lung, gonads, pancreas, salt gland, spleen, thyroid, thymus and urinary bladder) were separately observed to locate macroscopic vascular lesions or egg masses. The skull was opened for the examination of the brain only in fresh carcasses. In poorly preserved carcasses (*n* = 58), whenever possible, the heart, major vessels, gastrointestinal tract, and spleen were considered for gross examination.

Tissue samples (adrenal gland, *n* = 16; gastrointestinal tract, *n* = 66; heart and major vessels, *n* = 37; kidney, *n* = 47; liver, *n* = 45; lung, *n* = 42; gonads, *n* = 18; pancreas, *n* = 15; salt gland, *n* = 3; spleen, *n* = 52; thyroid, *n* = 15; thymus, *n* = 44; urinary bladder, *n* = 8) were collected, stored in 10% neutral buffered formalin, embedded in paraffin and sectioned at 4 μm for histological observation. The sections were stained with haematoxylin and eosin (HE) and observed under light microscopy (Olympus BX40F-3, Tokyo, Japan).

### Parasitological analyses

Stool samples (*n* = 168) and organs (gastrointestinal tract, *n* = 126, spleen, *n* = 64, heart and major vessels, *n* = 140) were collected according to carcass conditions and thoroughly analysed for the presence of blood flukes and their eggs.

Faecal samples were collected from the terminal portion of the intestine in all animals. An aliquot of each sample was stored at -20 °C for molecular analysis, while another aliquot (2–5 g) was submitted to qualitative copromicroscopic analysis to search for eggs. A common centrifugal sedimentation/flotation technique was applied using a high-density solution (sodium nitrate, sodium thiosulphate and sucrose/1.450).

The gastrointestinal tract and serosal vessels were longitudinally opened and examined both grossly and under a dissecting microscope to detect eggs, adult worms, and related lesions.

A pre-weighed portion (2 g) of splenic tissue was diced and homogenized by a blender in tap water. The obtained fluid was centrifuged in a tube at 2000× *rpm* for 5 min. After removal of the supernatant, a high-density solution (sodium nitrate, sodium thiosulphate and sucrose/1.450) was added and mixed with the sediment until the tube was filled; a coverslip was left over the tube for a few minutes and finally observed under a light microscope (10×) for the detection of spirorchiid eggs.

The heart chambers and major vessels (left and right aortas, brachiocephalic trunk and pulmonary arteries) were opened through longitudinal sectioning and rinsed with tap water. The washes were then submitted to sedimentation in conic flasks, and the sediment was observed under a stereomicroscope. Adult flukes were collected, counted and fixed in 70% ethanol for identification. The parasites were clarified in Amman’s lactophenol or stained with Semichon’s acid carmine and mounted in Canada balsam. The morphometric characteristics were studied under a light microscope by a calibrated eyepiece micrometre (Olympus, ACH 40X-2) and compared with descriptions in the literature [13, 24]. Spirorchiid eggs detected in faeces and spleen were measured under a light microscope and classified as type 1, 2 or 3 depending on the presence and number of lateral processes according to Wolke et al. [1].

The concordance between the different parasitological methods used to diagnose spirorchiid infection (i.e. qualitative copromicroscopic analysis, search for eggs in the spleen and observation of adult flukes in the cardiovascular system) was calculated as the number of samples with identical results divided by the total number of samples commonly examined (% concordance). The data were also evaluated using kappa-type statistics [28], which express the proportion of agreement beyond chance and provide a value (parameter *k*) ranging from 0 (no agreement) to 1 (perfect agreement).

### Genetic analysis of *Caretta caretta* hosts

To test the origin of the infected animals, a small amount (5 mm) of muscle tissues was collected from positive specimens. DNA was extracted using a QIAamp® DNA Mini and Blood Mini Kit (Qiagen GmbH, Hilden, Germany). A fragment of 815 bp of the mtDNA encompassing the control region (D-loop) was amplified by PCR using the primers LCM15382/H950 [29]. PCR conditions included an initial denaturing step at 94 °C for 3 min, followed by 36 cycles of 94 °C for 30 s, 52 °C for 30 s and 72 °C for 30 s, with a final step at 94 °C for 5 min. Amplification products were purified using a QIAquick PCR Purification Kit (Qiagen). A BigDye Terminator v3.1 Cycle Sequencing Kit (Applied Biosystems, Foster City, California) and the same primers as those used in the first PCR were used in the sequencing reactions. Purification was carried out with an Agencourt CleanSEQ Dye Terminator Removal Kit (Beckman Coulter, Cassina De’Pecchi, Italy) and loaded onto an ABI Prism™ 3130 Genetic Analyzer (Applied Biosystems). Sequences were analysed, and base called using DNA Sequencing Analysis Software version 5.1 (Applied Biosystems). The multiple alignment programs included in the package Vector NTI version 9.1 (Invitrogen, Carlsbad, California) was used to align the sequences. D-loop haplotypes were classified by comparison to the mtDNA sequences available in GenBank and deposited in the Archie Carr Center for Sea Turtle Research database (ACCSTR; http://accstr.ufl.edu/files/cclongmtdna.pdf).

### Molecular characterization of the parasites

Parasite DNA was extracted using a NucleoSpin® Tissue Kit (Macherey-Nagel, Duren, Germany) for adult flukes and using a PSP® Spin Stool DNA Kit (Invitek GmbH, Berlin, Germany) for stool samples that were positive in the copromicroscopic analysis.

The internal transcribed spacer 2 (ITS2) region of the rDNA was amplified using the primers described by Stacy et al. [30]. Amplification was performed by a standard PCR followed by a semi-nested PCR. The forward primer SPIR1 (5′-GAG GGT CGG CTT ATT ATC TAT CA-3′) and the reverse primer SPIR2 (5′-TCA CAT CTG ATC CGA GGT CA-3′) were used in the standard PCR. The forward primer SPIR1 and the reverse primer HLC4 (5′-TCA CAT CTG ATC CGA GGT CA-3′) were used in the semi-nested PCR. The PCR reactions were performed with a 30 μl reaction volume composed of 1–3 μl DNA, 2 mM MgCl_2_, 0.2 mM dNTPs (MBI Fermentas, Darmstadt, Germany), 1× PCR buffer, 0.5 μM each of the forward and reverse primer, and 1 U Platinum *Taq* DNA Polymerase (Invitrogen), with the remainder of the volume composed of sterile water. Cycling conditions included an initial activation step at 95 °C for 5 min., followed by 35 cycles of 94 °C for 30 s, 56 °C for 30 s, and 72 °C for 30 s, with a final extension step of 72 °C for 10 min.

The 28S region of the rDNA was amplified using the primers described by Olson et al. [31]: LSU-5 (5′-TAG GTC GAC CCG CTG AAY TTA AGCA-3′) and 1500R (5′-GCT ATC CTG AGG GAA ACT TCG-3′). The PCR reactions were performed in a 30 μl reaction volume composed of 3–5 μl DNA, 2.5 mM MgCl_2_, 0.2 mM dNTPs (MBI Fermentas, Germany), 1× PCR buffer, 0.8 μM each of forward and reverse primer, 1 M Betaine solution, and 1.5 U Platinum *Taq* DNA Polymerase (Invitrogen), with the remainder of the volume composed of sterile water. Cycling conditions included an initial activation step at 95 °C for 4 min, followed by 35 cycles of 94 °C for 60 s, 56 °C for 60 s, and 72 °C for 120 s, with a final extension step at 72 °C for 4 min.

All positive samples from PCR were sequenced by the Macrogen company (Macrogen Europe, Amsterdam, the Netherlands). Single electropherograms were analysed with the software ChromasPro version 2.4.3 (Technelysium Pty Ltd., South Brisbane, Australia).

The consensus sequences were assembled with the SeqMan program (DNASTAR package, Lasergene). The new nucleotide sequence data reported in this paper are available in the GenBank, EMBL and DDBJ databases (accession numbers: LT617052, *H. mistroides* ITS2 and LT882715, *H. mistroides* 28S; LT617053, *Neospirorchis* sp. ITS2 and LT882716 *Neospirorchis* sp. 28S; see Additional file 1: Table S1).

### Phylogenetic analysis

The nature of the newly generated sequences was confirmed by a BLAST search [32], performed against the non-redundant nucleotide database in GenBank. This search also allowed us to identify the orthologous sequences obtained for spirorchiids and species of closely related fluke families [31] (Additional file 1: Table S1).

The family Spirorchiidae is a paraphyletic group with respect to the Schistosomatidae [33]. Thus, we created two data sets containing all available ITS2 and 28S sequences of the family Spirorchiidae (Additional file 1: Table S1). We also included sequences of members of the family Schistosomatidae and species belonging to the family Aporocotylidae. These latter species were used as outgroups in the phylogenetic analyses.

After downloading the sequences listed in Additional file 1: Table S1, they were aligned and successively trimmed while considering the coverage of different portions of the evaluated markers. The trimming served to minimize the amount of missing data. The ITS2 and 28S datasets were aligned with the MAFFT program [34], which is available at the EBI website [35]. The multiple alignments were then imported into the MEGA 6 program [36] for analyses. The ITS2 alignment was 451 nt positions long, while the 28S alignment encompassed 700 nt positions.

The phylogenetic analyses were performed according to the maximum likelihood (ML) and Bayesian inference (BI) methods [37]. The IqTree program (version 1.5.4) was used for ML analyses [38]. One hundred independent tree searches were performed to avoid entrapment in local optimal trees. The molecular evolution models selected by the IqTree program [39] were as follows: TVM + I + G4 for the 28S dataset and GTR + I + G4 for the ITS2 dataset. The statistical support for tree topologies was computed by performing 10,000 ultrafast bootstrap replicates [40]. The program MrBayes (version 3.2.6) was used for BI analyses [41]. Two simultaneous runs, each of four chains, were performed in all analyses. Each run consisted of 1,000,000 generations, and trees were sampled every 100 generations (trees generated = 2 × 10^4^). Stationarity was considered reached when the average standard deviation of split frequencies was less than 0.005. ‘Burn-in’ was very stringent, and only the last 2000 generated trees were used to compute the majority-rule posterior consensus trees. The evolutionary model applied in Bayesian analyses was the GTR + I + G [37]. Pairwise distances among sequences were calculated with the MEGA 6 program [36].

## Results

### Parasitological analyses

Spirorchiidiasis was observed in 28 of the 168 examined animals (16.7%). The results of the analyses used to detect and identify adult flukes and spirorchiid eggs in the cardiovascular district, stool samples and spleen are reported in Table 1.

**Table 1:** Biometric data and results of analyses for *Caretta caretta* positive for spirorchiid infection

ID	SCL (cm)	BCS	Sex	mtDNA haplotype	Examination for spirorchiid eggs			Examination for adult flukes^a^	
					Stool samples		Spleen samples		
					Egg type	Molecular identification^b^	Egg type	Detection and morphological identification	Molecular identification
23451	83	3	F	CC - A2.1	Type 1	*H. mistroides*	nd	nd	nd
27177	55	1	F	CC - A2.1	Type 1	*H. mistroides*	Type 1	Negative	nd
32010	55	2	M	CC - A2.1	Type 1	*H. mistroides*	nd	nd	nd
55775	40	2	M	CC - A2.1	Type 1	*H. mistroides*	nd	Negative	nd
56275	71	2	F	CC - A2.1	Type 1	Negative	nd	Negative	nd
56284	52	2	F	CC - A2.8	Type 1	*H. mistroides*	Type 1	Negative	nd
56300	61	2	F	CC - A2.1	Type 1	*H. mistroides*	Type 1	*Hapalotrema* sp.	*H. mistroides*
56301	49	2	F	CC - A2.1	Type 1	*H. mistroides*	Negative	Negative	nd
56365	53	2	M	CC - A2.1	Type 1	*H. mistroides*	Negative	Negative	nd
57391	nd	1	F	CC - A2.1	Type 3	*Neospirorchis* sp.	Negative	Negative	nd
58955	75	2	F	CC - A2.1	Type 1	*H. mistroides*	Type 1	Negative	nd
61468	61	1	M	CC - A2.1	Type 1 + 3	Negative	Type 1	Negative	nd
61467	45	2	F	CC - A2.1	Type 1 + 3	*H. mistroides* and *Neospirorchis* sp.	Type 1	*Hapalotrema* sp.	*H. mistroides*
61733	35	3	F	CC - A2.1	Type 1	Negative	Type 1	Negative	nd
61918	69	2	M	CC - A2.1	Type 3	*Neospirorchis* sp.	Type 3	Negative	nd
62452	64.5	2	F	CC – A32.1	Type 1 + 3	*H. mistroides* and *Neospirorchis* sp.	nd	Negative	nd
62955	68	2	F	CC - A2.1	Type 1	*H. mistroides*	Type 1	Negative	nd
63355	77	2	M	CC - A2.1	Type 1 + 3	*H. mistroides* and *Neospirorchis* sp.	Type 1	*Hapalotrema* sp.	*H. mistroides*
62953	51.5	2	F	CC - A2.1	Type 1 + 3	*H. mistroides* and *Neospirorchis* sp.	Type 1	*Hapalotrema* sp.	*H. mistroides*
63475	63	1	F	CC - A2.1	Type 1	*H. mistroides*	Type 1	*Hapalotrema* sp.	*H. mistroides*
63477	59	2	M	CC - A2.1	Type 1	*H. mistroides*	Type 1	Negative	nd
64893	43	2	M	nd	Type 1	*H. mistroides*	Type 1	Negative	nd
64985	51	2	M	nd	Type 1	*H. mistroides*	nd	*Hapalotrema* sp.	*H. mistroides*
65004	35	3	M	nd	Type 1	*H. mistroides*	Type 1	nd	nd
65734	58	2	F	nd	Type 1 + 3	*H. mistroides*	nd	Negative	nd
65735	73	1	F	nd	Type 1 + 3	*H. mistroides* and *Neospirorchis* sp.	Type 1	Negative	nd
65000	80	2	F	nd	Type 1	*H. mistroides*	Type 1	Negative	nd
65225	44	2	F	nd	Type 1 + 3	*H. mistroides* and *Neospirorchis* sp.		Negative	nd

*Abbreviations*: *ID* host identification code, *SCL* straight carapace length, *BCS* body condition score, *nd* not determined

^a^ In the cardiovascular system

^b^Molecular analyses in mixed infections were performed on eggs isolated directly from the intestinal wall

Adult flukes were found inside the heart and at the beginning of the major vessels in six animals. Because of the poor conditions of the specimens, the morphological approaches only permitted identification of the parasites as members of *Hapalotrema* based on the presence of multiple testes anterior and posterior to the ovary and terminal genitalia.

Copromicroscopic examination proved positive results for spirorchiid eggs in 28 cases; type 1 and 3 eggs were found in 26 and 10 animals, respectively (15.5 and 6.0%), and there were 8 cases of mixed infection. The analyses carried out on spleen samples revealed the presence of spirorchiid eggs in 17 cases; type 1 and 3 eggs were observed in 16 and one sample(s), respectively. Of 28 animals found positive in the copromicroscopic exam, 14 specimens had eggs (i.e. type 1, type 3 or both) that were detected on the gastrointestinal walls. All animals with eggs in the spleen and adults in the cardiovascular system were also positive in the copromicroscopic analysis. Table 2 shows the comparisons among the performed techniques regarding the percentage of concordance and level of agreement (parameter *k*). The copromicroscopic analysis was in excellent agreement with the evaluation of eggs in the spleen (*n* = 64), whereas the correspondence with the evaluation of adults in cardiovascular system was only fair (*n* = 140).

**Table 2:** Comparison between copromicroscopic examination and evaluation of spirorchiids in the spleen and cardiovascular system

		Coproscopic examination (eggs)				
		Neg	Pos	Total	% concordance	Parameter *k*
Spleen examination (eggs)	Neg	44	3	47	95.3	0.886
	Pos	0	17	17		
	Total	44	20	64		
Cardiovascular system (adults)	Neg	116	18	134	87.1	0.356
	Pos	0	6	6		
	Total	116	24	140		

*Abbreviations*: *Neg* negative sample, *Pos* positive sample

### Genetic analyses of *Caretta caretta* hosts

Twenty-one of 28 positive *C. caretta* were genetically analysed. Sequences of the same length (815 bp) were obtained for all the samples (Table 1). The comparison of these sequences with those registered in the databases for *C. caretta* led to the identification of three already known D-loop haplotypes. CC-A2.1 (GenBank: EU179445) was the most frequent haplotype and was detected in 19 host specimens (91%). One individual (4.5%) collected in 2013 exhibited the haplotype CC-A2.8 (FM200217), while the haplotype CC-A32.1 (JF837822) was recorded for one turtle (4.5%) collected in 2014.

### Anatomopathological findings

Body condition score of the positive animals is shown in Table 1.

**Fig. 1:**
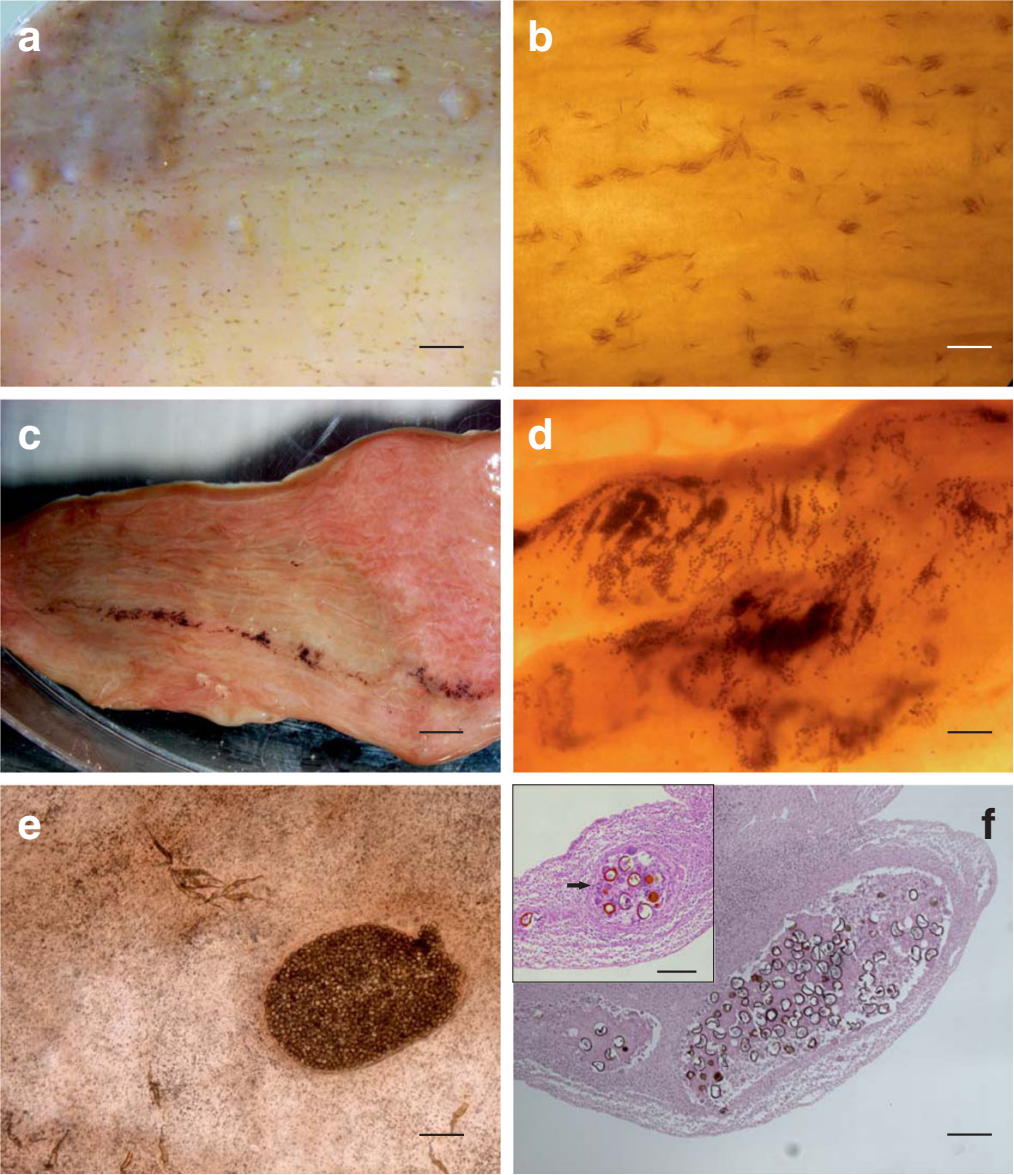
Intestinal lesions in *Caretta caretta* associated with *Hapalotrema mistroides* and *Neospirorchis* sp. Different pattern of distribution on the intestinal district are easily visible for the two genera. Type 1 eggs are scattered on the intestinal wall, grossly looking as small brownish spots (**a**) composed of a low number of fusiform eggs as revealed by stereomicroscopy (**b**). Big masses of type 3 eggs are visible on the intestinal mucosa as elongated black serpiginous stripes (**c**), formed by thousands of elements (**d**). Different patterns of distribution are easily visible in this case of mixed infection, in which type 3 eggs appear like grouped in a cyst like structure (**e**). Multifocal granulomatous enteritis (**f**; HE) with multinucleated giant cells (black arrow) surrounding a core of eggs and necrotic debris is detectable in intestinal sections with minimal fibrotic reaction (inset). *Abbreviation*: HE, haematoxylin and eosin. *Scale-bars*: **a**, 0.5 cm; **b**, 1 mm; **c**, 0.37 cm; **d**, 560 μm; **e**, 350 μm; **f**, 150 μm (inset: 120 μm)

Spirorchiid eggs were detected in the intestinal mucosa and submucosa only in animals found to be positive by copromicroscopic exam. The distribution pattern of the eggs on the intestinal walls was macroscopically different for the two types. Type 1 eggs were arranged in small clusters or disseminated as single elements in the mucosal layer. Type 3 eggs were arranged in highly visible, serpiginous masses included in the mucosal layer or, less frequently, as large clusters with a cyst-like appearance and black (Fig. 1).

Egg clusters were surrounded by mild granulomatous inflammation with multinucleated giant cells, rare mixed inflammatory cells, and a thin fibrous capsule. Single elements and small groups of eggs (*n* = 3–5) were not associated with any inflammatory reaction. Granulomas were seen in the spleen, lung, thymus, and pancreas surrounding small, multifocal groups of eggs. Isolated eggs were also observed in the gastric walls, liver, heart, kidney, adrenal gland, salt glands, and urinary bladder wall.

**Fig. 2:**
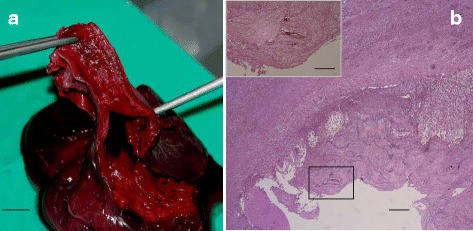
**a** Heart and great vessels. Small proliferative plaques are visible on the supravalvular region on the intima of a great vessel in a specimen of *C. caretta* infected with *Hapalotrema mistroides*. **b** Heart, atrium (HE). Raised irregular plaques of severe chronic endocarditis and extensive fibroplasia with multifocal areas of undetermined mesenchymal cells surrounding some eggs (inset). *Abbreviation*: HE, Haematoxylin and eosin. *Scale-bars*: **a**, 1 cm; **b**, 290 μm (inset: 135 μm)

Mild to moderate arteritis was observed in four of six animals from which adult parasites were isolated. The beginning of the major vessels was the most affected section (Fig. 2). Small proliferative plaques were grossly visible on the intima with rough irregular surface. At histology, fibro-muscular proliferations were visible inside the intima of the inflamed vessels; isolated eggs were frequently seen more deeply in the vessel walls without any associated inflammatory processes. In one case, extensive sub-endothelial inflammatory infiltrate was observed in association with multiple degenerated eggs.

### Molecular identification of parasites and phylogenetic analysis

The ITS2 sequences were obtained for twenty-three faecal samples positive for type 1 eggs and six adult flukes (one from each positive animal). All ITS2 sequences were identical. A BLAST search identified the ITS2 sequence from a specimen of *H. mistroides* (*H. mistroides* GU937893, Additional file 1: Table S1) as the closest relative to the ITS2 sequences in the current study. Pairwise comparisons among the new sequences and *H. mistroides* A ITS2 showed an identity of 99.63% (i.e. one base A *vs* G was different over 270 bases of the alignment). The different base is located near the 3′ end, a portion not included in the phylogenetic analysis. Thus, the newly generated sequences were assigned to *H. mistroides* (see below). The ITS2 sequences obtained from eight faecal samples positive for type 3 eggs were found to be identical. These sequences were compared with data available in GenBank and were found to be identical to those obtained from *Neospirorchis* sp. Neogen11 (KU601335) (Additional file 1: Table S1).

The 28S partial sequences obtained from four adults of *H. mistroides* were identical. Similarly, the 28S sequences obtained from five samples of *Neospirorchis* sp. eggs were identical. We obtained a single haplotype for both taxa.

**Fig. 3:**
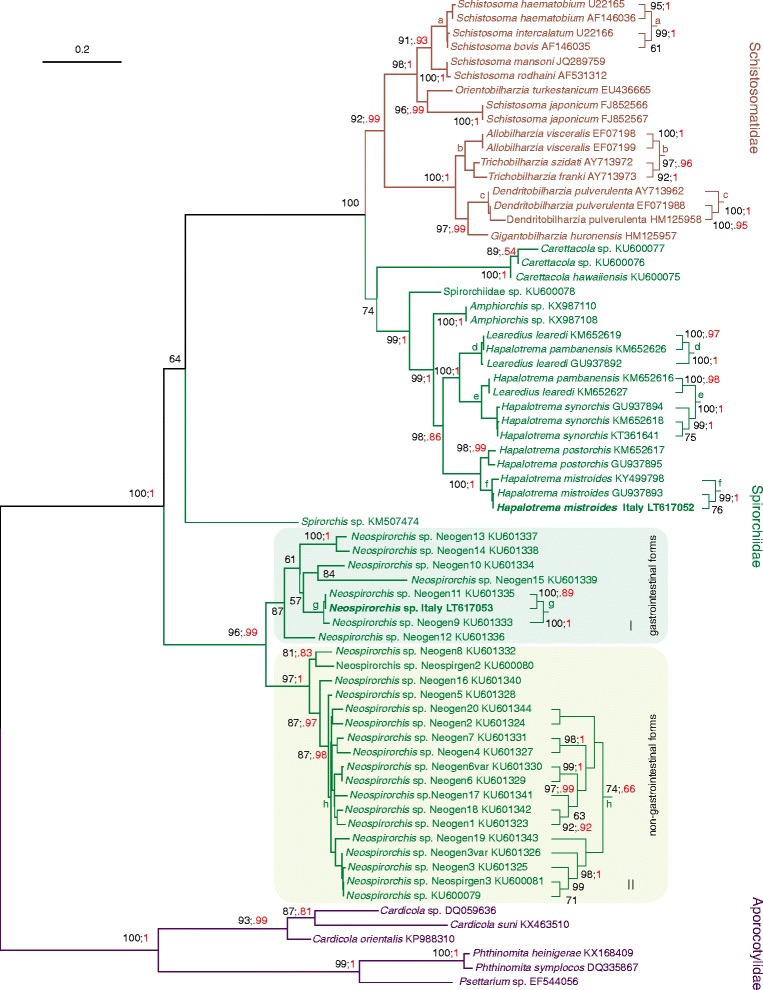
Phylogenetic analysis of spirorchiid flukes based on ITS2 sequences. The ML tree (-ln = 6007.5517) was computed with IQ-TREE program. The scale-bar represents 0.2 substitutions/state change per position. Numbers in black represent ultrafast bootstrap values (> 50%) expressed in percent; numbers in red refer to Bayesian Inference posterior probabilities presented in a compressed way (e.g. 1 instead of 1.00; .95 instead of 0.95) to allow a better readability of the figure

The ML tree obtained from the ITS2 alignment is presented in Fig. 3. The BI consensus tree exhibited a topology that was mostly congruent except for the arrangement of the *Neospirorchis* clade (see below). The family Spirorchiidae was paraphyletic with respect to the Schistosomatidae. Two major clades occurred within Spirorchiidae. The first clade included exclusively taxa belonging to the genus *Neospirorchis*. The second clade contained the other analysed spirorchiid taxa and was a sister group of the Schistosomatidae. The *Neospirorchis* clade received strong bootstrap and BI posterior probability support. *Neospirorchis* sp. Italy (LT617053) was placed within the *Neospirorchis* clade as a sister taxon of *Neospirorchis* sp. Neogen 11 (KU601335). In the ML tree, two clades (I and II) could be identified within the genus *Neospirorchis*. Both clades received bootstrap corroboration. The phylogenetic relationships among the different *Neospirorchis* taxa were well resolved and received bootstrap corroboration in many cases. However, clade I was not found in the BI consensus tree. *Hapalotrema mistroides* Italy (LT617052) formed a clade with two other specimens assigned to the same species. This group received strong statistical corroboration by bootstrap and BI posterior probability values. Samples of *H. mistroides* were the sister group of *Hapalotrema postorchis* specimens, a phylogenetic relationship supported by both bootstrap and BI posterior probability values. *Hapalotrema pambanensis* and *Learedius learedi* were not monophyletic.

**Fig. 4:**
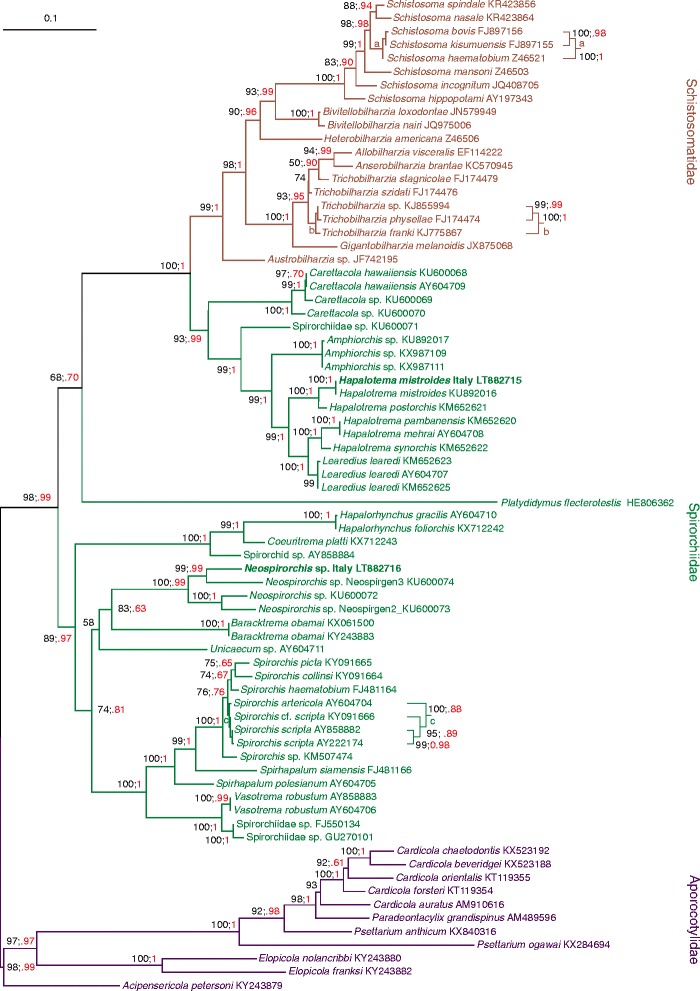
Phylogenetic analysis of spirorchiid flukes based on 28S sequences. The ML tree (-ln = 10,754.3072) was computed with IQ-TREE program. The scale-bar represents 0.1 substitutions/state change per position. Numbers in black represent ultrafast bootstrap values (> 50%) expressed in percent; numbers in red refer to Bayesian Inference posterior probabilities presented in a compressed way (e.g. 1 instead of 1.00; .95 instead of 0.95) to allow a better readability of the figure

The maximum likelihood tree obtained from the 28S multiple alignment is presented in Fig. 4. The BI consensus tree exhibited a fully congruent topology (data not shown). Most of the nodes received support by bootstrap and BI posterior probability values. The family Spirorchiidae was paraphyletic with respect to Schistosomatidae. Two main clades could be recognized within the spirorchiid flukes. A clade containing mostly species of the genera *Carettacola*, *Amphiorchis*, *Hapalotrema* and *Learedius* was a sister group of the Schistosomatidae. Within this clade, *L. learedi* (represented by three sequences) and *H. pambanensis* (represented by two sequences) were monophyletic. Indeed, *Hapalotrema mehrai* Rao,1976 is a junior synonym of *H. pambanensis* (Additional file 1: Table S1). The second major clade included various spirorchiid taxa. *Neospirorchis* sp. Italy (LT882716) grouped with the other taxa of the genus *Neospirorchis*. Similarly, *H. mistroides* Italy (LT882715) was a sister taxon to *H. mistroides* (KU892016).

The average p-distance among the ITS2 sequences of the *Neospirorchis* taxa was 0.128 ± 0.075. The p-distances ranged from 0 [*Neospirorchis* sp. Italy (LT617053) *vs* *Neospirorchis* sp. Neogen11 (KU601335)] to the maximum value of 0.291 observed in *Neospirorchis* sp. Neospirgen2 (KU600080) *vs* *Neospirorchis* sp. Neogen15 (KU601339). The average p-distance among taxa belonging to the *Hapalotrema* + *Learedius* clade was 0.122 ± 0.064. The range varied from 0 [*H. mistroides* Italy (LT617052) *vs* *H. mistroides* (GU937893)] to the maximum value of 0.195 observed in the pair *H. mistroides* (KY499798) *vs* *H. synorchis* (KM652618). Finally, within the genus *Schistosoma*, the average p-distance was 0.110 ± 0.072, while values ranged from 0 [*Schistosoma japonicum* (FJ852567) *vs* *Schistosoma japonicum* (FJ852566)] to 0.188 [*Schistosoma japonicum* (FJ852566) *vs* *Schistosoma haematobium* (U22165)].

## Discussion

### Prevalence data and ecological considerations

Few studies on spirorchiidiasis in loggerhead turtles have been carried out [1, 4, 15, 16, 42]; these studies involve a small number of animals and apply different methodologies for parasite detection. Only two surveys carried out in Florida can be considered for a comparison with the current study due to having a similar diagnostic approach and sampling effort [1, 11]. The prevalence of infection observed in our study for both *Hapalotrema* sp. and *Neospirorchis* spp. appears to be lower than that recorded in the two previous studies. In the most recent survey [11], the high percentage of loggerhead sea turtles infected by both *Hapalotrema* spp. and *Neospirorchis* spp. (96 and 77%, respectively) demonstrated a wide diffusion of infection in the north-western Atlantic. In the present study, spirorchiidiasis does not appear to be widespread among loggerhead turtles feeding in the northern Adriatic grounds (prevalence of 15.5 and 6.0% for *Hapalotrema* and *Neospirorchis*, respectively). There was one case of infection by *H. mistroides* observed in one loggerhead turtle stranded along the coast of central-western Italy [22]; however, other surveys performed in last few decades did not detect these parasites in *C. caretta* in the central or eastern Mediterranean areas [43–45]. These results may be partly due to different research methodologies or to minor sampling effort. Nevertheless, this finding supports the hypothesis that spirorchiidiasis did not raise the attention of researchers for frequent and serious patterns of infection in the region, which differs from the descriptions for North Atlantic waters [15]. The differences in the spread of infection may be due to environmental factors, including the presence and distribution of the intermediate hosts. Involvement of a gastropod intermediate host in the life-cycle of marine spirorchiids has been already demonstrated [16, 30].

The detection of adult flukes in a few cases may have been due to poor sample quality, the small body size of these trematodes and the tendency, especially for *Neospirorchis* spp., to inhabit small vessels, as already shown by other authors [3, 4]. Therefore, it is commonly recognized that the diagnosis of spirorchiidiasis cannot rely only on the detection of adult specimens. Spirorchiid eggs have greater resistance to decomposition than adults both in carcasses and in the environment [46]. Faecal examination has been used to detect infections in sea turtles [1], although it is considered a diagnostic method lacking in specificity and sensitivity [25, 47]. In this study, copromicroscopic examination showed excellent agreement (*k* = 0.886) with the method used for searching for eggs in the spleen, a common site of egg deposition due to its role as a blood filtering organ [2]. The presence of eggs in the faeces is related to their migration through the intestinal walls and entrance into the gut lumen [12, 47], but not all species have the primary route of elimination via the gastrointestinal tract in all host species [4, 47]. Our positive results from the copromicroscopic exams were often associated with the presence of both *Hapalotrema mistroides* and *Neospirorchis* sp. eggs in the intestinal mucosa; therefore, copromicroscopic examination remains a valid and noninvasive diagnostic method in the case of infection by *Hapalotrema mistroides* and *Neospirorchis* spp. in *C. caretta* and is also feasible for use with live animals. Copromicroscopic analysis has additional limitations, such as recent infections (immature specimens) or low parasite burden, that could produce false negative results, and since different spirorchiid genera have similar eggs, molecular approaches remain essential for certainty in identification.

Our genetic analyses were performed on positive hosts to test the hypothesis that spirorchiidiasis was acquired inside the Mediterranean Basin. Two recent reports [16, 22] demonstrated the presence of *H. mistroides* infection in loggerhead sea turtle specimens from the western Mediterranean and the Tyrrhenian Sea. However, no genetic analyses were done in those studies, and considering that both Atlantic and Mediterranean turtles can be found in the western part of the basin, uncertainty about their origins remains. The three D-loop haplotypes found in the present study are common in loggerhead sea turtles from the Mediterranean Basin [48]; haplotype CC-A2.1 is the most frequent in all Mediterranean rookeries [48] and is found at low frequencies in Atlantic colonies [49]. Haplotype CC-A2.8 was observed in loggerhead turtles nesting in Crete [48] and feeding in Ionian waters [50], while haplotype CC-A32.1 is private of Greek nesting colonies [48]. The presence of individuals from these two Mediterranean nesting colonies in Adriatic waters has been documented by genetic studies [50–52] as well as by satellite tracking studies [53, 54]. Therefore, the positive animals seem to belong to the stock usually encountered in the northern Adriatic waters. Loggerhead sea turtles of the Mediterranean, particularly those of Greek origin, show intra-Mediterranean migratory pathways and select neritic feeding areas inside the basin to which they show strong fidelity [55], sometimes sharing the same foraging area with turtles of Atlantic origin (i.e. the Gulf of Gabès, Tunisia [53]). These points enhance the probability that the turtles (at least those carrying Greek haplotypes) acquired the infection inside the Mediterranean Sea along their migratory routes.

### Pathogenic impact

Spirorchiidiasis in sea turtles from the Atlantic and Pacific Oceans has been found to be causal or contributory to death in many cases [3, 11, 16, 56]. In the Mediterranean Sea, except for scarce historical data, there is only one case report of a free-ranging loggerhead turtle [22] in which severe lesions by spirorchiids likely contributed to the death of the animal. Nevertheless, in the present study spirorchiidiasis never affected the general health status of the hosts and represented an occasional finding during necropsy. Most of the positive turtles were in a good or excellent nutritional condition, and only mild lesions were observed.

Two patterns of lesions were observed for type 1 and type 3 eggs in the intestine and were similar to that described by Stacy et al. [4] in *C. caretta* for *Hapalotrema* spp. and *Neospirorchis* spp., respectively. In *Hapalotrema*-infected hosts, the severity of the granulomatous reaction to egg masses was higher, with transmural and prominent lesions visible both on the intestinal mucosa and on the subserosal vessels. In our study, the mucosal and (rarely) submucosal layers were affected, and no lesions were evident from the external surface of the intestinal walls.

Spirorchiids are known to produce serious pathological lesions in the circulatory system of the host. Arteritis, aneurysms and disseminated thrombi are described in various districts in *C. caretta* infected by *H. mistroides* and *Neospirorchis* spp. [1, 4]. Some differences in the distribution of adult flukes and lesions are reported depending on the infecting species and genotypes [11]. In our study, most of the positive turtles had no lesions in the cardiovascular system except for a few cases with mild to moderate alterations at the emergence of the major vessels where adult specimens of *H. mistroides* were collected. Associations between *Hapalotrema* spp. (including *H. mistroides*) and endarteritis was already shown by Stacy et al. who also found parasites attached to the lesions [4].

### Molecular and phylogenetic findings

The specimens assigned to the genus *Neospirorchis* sequenced in our study shared the same ITS2 haplotype, *Neospirorchis* sp. Italy (LT617053). The latter was identical to the *Neospirorchis* sp. Neogen11 (KU601335) sequence obtained from a specimen previously isolated from the gastrointestinal tract of a *C. caretta* specimen in Florida [11]. Similarly, all the specimens assigned to *H. mistroides* Italy (LT617052) had identical ITS2 sequences that differed from *H. mistroides* (GU937893) by a single base (A *vs* G) located outside of the aligned portion.

The 28S sequences of *H. mistroides* Italy LT882715 and *H. mistroides* KU892016 were also identical (Fig. 4). The high levels of sequence similarity were mirrored by the phylogenetic results (Figs. 3 and 4), where newly generated sequences grouped with the orthologous counterparts obtained from congeneric (*Neospirorchis*) or conspecific specimens (*H. mistroides*). Multiple sequences were available for both *Neospirorchis* spp. and *H. mistroides*. If our sequences do not belong to the genus *Neospirorchis*/ *H. mistroides*, these taxa become paraphyletic in the ITS2 analysis, a situation that is untenable. Thus, the phylogenetic analysis allowed the straightforward generic/specific assignment of the new sequences.

The level of molecular variation observed among the *Neospirorchis* ITS2 sequences was higher than that observed among the species included in the *Hapalotrema* + *Learedius* clade or within the genus *Schistosoma*. These results support the view that the *Neospirorchis* flukes parasitizing sea turtles belong to at least two distinct species.

The analysis performed on the ITS2 of *H. pambanensis* and *L. learedi* suggested a non-monophyly of these taxa. In our opinion, these results must be regarded as an artifact due to a mislabelling of the sequences submitted to GenBank. In favour of our hypothesis, there are the following considerations. First, Chapman et al. [25] generated and used this set of sequences in a previous phylogenetic analysis where both species were monophyletic. Unfortunately, they did not present the accession numbers of the sequences in their phylogenetic tree. Secondly, in our 28S tree, both taxa are also monophyletic. Thirdly, it is unrealistic that two distinct species would present identical ITS2 sequences considering the level of variation also observed in the present study for these molecular markers.

## Conclusions

In this study, eggs or adults from two species of spirorchiids, *H. mistroides* and *Neospirorchis* sp., were observed in single or mixed infections and were identified by both morphological and molecular approaches. *Hapalotrema mistroides* was already described in loggerheads stranded along Egyptian [18, 19] and Italian coastlines [17, 22], whereas the genus *Neospirorchis* is described in this basin for the first time. Our study noted the importance of molecular approaches and phylogenetic studies as a good complement for resolving problems related to poorly preserved specimens and to provide evidence of cryptic speciation within the currently recognized spirorchiid species, as previously suggested by other authors [11, 25]. Although spirorchiidiasis is one of the oldest observations in sea turtle literature [17], this study provides prevalence data with pathological features of the infection for the first time inside the Mediterranean Basin and updates our knowledge on the species present. Low prevalence and mild lesions were observed in the samples, leading to the conclusion that spirorchiidiasis does not represent a cause of severe morbidity or mortality for turtles in the region. Data on *C. mydas*, also a resident species in the Mediterranean, are completely lacking, and studies performed in the western Mediterranean included a limited number of loggerhead sea turtles. Therefore, further investigations are necessary for more thorough knowledge of the ecopathology and spread of spirorchiidiasis in the Mediterranean Sea. Considering the results of the genetic analysis carried out on positive turtles and the current knowledge of the migratory routes of Mediterranean turtles, the hypothesis that infection by both *Hapalotrema* and *Neospirorchis* was acquired inside the Mediterranean Basin seems to be supported. The identity of the ITS2 sequences of *Neospirorchis* sp. Italy and Neogen-11 from Florida suggest the possibility that the infection was transmitted by Atlantic turtles to Mediterranean turtles through an infected intermediate host in a shared feeding ground. If this hypothesis is confirmed, this would highlight the importance of studying the roles of these areas as ecological key points for the spill over of pathogens between the two populations.

## Additional file

Additional file 1: Table S1. List of fluke taxa analysed in the present study with GenBank accession numbers, sources, hosts and localities. (DOCX 67 kb)
