# Supplementary material for: Cardiovascular flukes (Trematoda: Spirorchiidae) in Caretta caretta Linnaeus, 1758 from the Mediterranean Sea
Source: Parasit Vectors. 2017 Oct 10;10:467. doi: 10.1186/s13071-017-2396-x (PMC5633879; doi:10.1186/s13071-017-2396-x)
Supplement: Additional file 1: Table S1. — List of fluke taxa analysed in the present study with GenBank accession numbers, sources, hosts and localities. (DOCX 67 kb) [file 13071_2017_2396_MOESM1_ESM.docx]

**Additional file 1: Table S1 List of fluke taxa analysed in the present study with GenBank accession numbers, sources, hosts and localities**

| **Taxon** |  | **28S** | | |  | **ITS2** | | |  | **Source** | **Host** | **Country** |
| --- | --- | --- | --- | --- | --- | --- | --- | --- | --- | --- | --- | --- |
| **Aporocotylidae Odhner, 1912** |  |  |  |  |  |  |  |  |  |  |  |  |
| *Acipensericola petersoni* Bullard, Snyder, Jensen & Overstreet, 2008 |  | KY243879 |  |  |  |  |  |  |  | P [1] | *Polyodon spathula* | Tennessee River (USA) |
| *Cardicola auratus* Holzer, Montero, Repullés, Sitjà-Bobadilla, Alvarez-Pellitero, Zarza & Raga, 2008 |  | AM910616 |  |  |  |  |  |  |  | P [2] | *Sparus aurata* | Valencia (Spain) |
| *Cardicola beveridgei* Nolan, Miller, Cutmore, Cantacessi & Cribb, 2014 |  | KX523188 |  |  |  |  |  |  |  | P [3] | *Lutjanus argentimaculatus* | Lizard Island, Queensland (Australia) |
| *Cardicola chaetodontis* Yamaguti, 1970 |  | KX523192 |  |  |  |  |  |  |  | P [3] | *Chaetodon rainfordi* | Heron Island, Queensland (Australia) |
| *Cardicola forsteri* Cribb, Daintith & Munday, 2000 |  | KT119354 |  |  |  |  |  |  |  | P [4] | *Neoamphitrite* sp. | Japan |
| *Cardicola orientalis* Ogawa, Tanaka, Sugihara & Takami, 2010 |  | KT119355 |  |  |  | KP988310 |  |  |  | P [4], P [5] | *Nicolea gracilibranchis/Thunnus thynnus* | Japan/Spain |
| *Elopicola franksi* Orélis-Ribeiro & Bullard, 2017 |  | KY243882 |  |  |  |  |  |  |  | P [1] | *Megalops atlanticus* | Gulf of Mexico (USA) |
| *Cardicola* sp. |  |  |  |  |  | DQ059636 |  |  |  | P [6] | *Siganus fuscescens* | Australia |
| *Cardicola suni* Yong, Cutmore, Miller, Wee & Cribb, 2016 |  |  |  |  |  | KX463510 |  |  |  | P [3] | *Chanos chanos* | Moreton Bay, Queensland (Australia) |
| *Elopicola nolancribbi* Bullard, 2014 |  | KY243880 |  |  |  |  |  |  |  | P [1] | *Elops saurus* | Gulf of Mexico (USA) |
| *Paradeontacylix grandispinus* Ogawa & Egusa, 1986 |  | AM489596 |  |  |  |  |  |  |  | P [7] | *Seriola dumerili* | Ushine, Kagoshima (Japan) |
| *Psettarium anthicum* Bullard & Overstreet, 2006 |  | KX840316 |  |  |  |  |  |  |  | P [8] | n.d. | n.d. |
| *Phthinomita heinigerae* Nolan, Cantacessi, Cutmore, Cribb & Miller, 2016 |  |  |  |  |  | KX168409 |  |  |  | P [9] | *Taeniamia fucata* | Australia |
| *Phthinomita symplocos* Nolan & Cribb, 2016 |  |  |  |  |  | DQ335867 |  |  |  | U [10] | *Siganus lineatus* | Australia |
| *Psettarium ogawai* Yong, Cutmore, Bray, Miller, Semarariana, Palm & Cribb, 2016 |  | KX284694 |  |  |  |  |  |  |  | P [11] | *Arothron reticularis* | Bali (Indonesia) |
| *Psettarium* sp. |  |  |  |  |  | EF544056 |  |  |  | U [12] | *Takifugu niphobles* | Japan |
| **Schistosomatidae Stiles et Hassall, 1898** |  |  |  |  |  |  |  |  |  |  |  |  |
| *Allobilharzia visceralis* Kolarova, Rudolfova, Hampl & Skirnisson, 2006 |  | EF114222 |  |  |  | EF071990 | EF071989 |  |  | P [13] | *Cygnus columbianus* | USA |
| *Anserobilharzia brantae* (Farr & Blankemeyer, 1956) |  | KC570945 |  |  |  |  |  |  |  | P [14] | *Branta canadensis* | Canada |
| *Austrobilharzia* sp. |  | JF742195 |  |  |  |  |  |  |  | P [15] | *Cerithidea cingulata* | Kuwait |
| *Bivitellobilharzia loxodontae* Vogel & Minning, 1940 |  | JN579949 |  |  |  |  |  |  |  | P [16] | *Loxodonta cyclotis* | Central African Republic |
| *Bivitellobilharzia nairi* (Mudaliar & Ramanujachar, 1945) |  | JQ975006 |  |  |  |  |  |  |  | P [17] | *Elephas maximus* | Nepal |
| *Dendritobilharzia pulverulenta* (Braun, 1901) |  |  |  |  |  | HM125958 | AY713962 | EF071988 |  | P [18], P [19], P [13] | *Gyraulus parvus/Gallus gallus/mergus merganser* | USA |
| *Gigantobilharzia huronensis* Najim, 1950 |  |  |  |  |  | HM125957 |  |  |  | P [18] | *Physa gyrina* | USA |
| *Gigantobilharzia melanoidis* Schuster, Aldhoun & O’Donovan, 2014 |  | JX875068 |  |  |  |  |  |  |  | P [20] | *Melanoides tuberculata* | United Arab Emirates |
| *Heterobilharzia americana* Price, 1929 |  | Z46506 |  |  |  |  |  |  |  | P [21] | n.d. | n.d. |
| *Orientobilharzia turkestanicum* (Skrjabin, 1913) |  |  |  |  |  | EU436665 |  |  |  | P [22] | Cashmere goat | China |
| *Schistosoma bovis* (Bilharz, 1852) |  | FJ897156 |  |  |  | AF146035 |  |  |  | P [23], P [24] | *Mastomys natalensis* | Nyabera Swamp, Kisumu (Kenya) |
| *Schistosoma haematobium* (Bilharz, 1852) |  | Z46521 |  |  |  | AF146036 | U22165 |  |  | P [21], P [24], P [25] | n.d. | Egypt |
| *Schistosoma hippopotami* Thurston, 1963 |  | AY197343 |  |  |  |  |  |  |  | P [26] | *Bulinus truncatus* | Lake Edward (Uganda) |
| *Schistosoma incognitum* Chandler, 1926 |  | JQ408705 |  |  |  |  |  |  |  | P [27] | Barbari goat | India |
| *Schistosoma intercalatum* Fisher, 1934 |  |  |  |  |  | U22166 |  |  |  | P [25] | n.d. | n.d. |
| *Schistosoma japonicum* (Katsurada, 1904) |  |  |  |  |  | FJ852567 | FJ852566 |  |  | P [28] | *Oncomelania upensis robertsoni* | Yunnan Province (China) |
| *Schistosoma kisumuensis* Hanelt, Brant, Steinauer, Maina, Kinuthia, Agola, Mwangi, Mungai, Mutuku, Mkoji & Loker, 2009 |  | FJ897155 |  |  |  |  |  |  |  | P [23] | *Dasymys incomtus* | Niabera Swamp, Kisumu (Kenya) |
| *Schistosoma mansoni* Sanbon, 1907 |  | Z46503 |  |  |  | JQ289759 |  |  |  | P [21], P [29] | n.d. | Kenya |
| *Schistosoma nasale* Rao, 1933 |  | KR423864 |  |  |  |  |  |  |  | P [30] | Snail | Nepal |
| *Schistosoma spindale* (Montgomery, 1906) |  | KR423856 |  |  |  |  |  |  |  | P [30] | Snail | Napal |
| *Schistosoma rodhaini* Brumpt, 1931 |  |  |  |  |  | AF531312 |  |  |  | P [31] | *Biomphalaria sudanica* | Homa Bay (Kenya) |
| *Trichobilharzia franki* Muller & Kimmig, 1994 |  | KJ775867 |  |  |  | AY713973 |  |  |  | P [32], P [19] | *Radix auricularia/lymnaea stagnalis* | England Hampshire (UK)/Czech Republic |
| *Trichobilharzia physellae* (Thalbot, 1936) |  | FJ174474 |  |  |  |  |  |  |  | P [33] | *Bucephala alveola* | USA |
| *Trichobilharzia* sp. |  | KJ855994 |  |  |  |  |  |  |  | P [34] | *Physa marmorata* | Brazil |
| *Trichobilharzia stagnicolae* (Thalbot, 1936) |  | FJ174479 |  |  |  |  |  |  |  | P [33] | *Stagnicola emarginata* | USA |
| *Trichobilharzia szidati* Neuhaus, 1952 |  | FJ174476 |  |  |  | AY713972 |  |  |  | P [33], P [19] | *Lymnaea stagnalis* | USA/ Czech Republic |
| **Spirorchiidae Stunkard, 1921** |  |  |  |  |  |  |  |  |  |  |  |  |
| *Amphiorchis* sp. |  | KU892017 |  |  |  |  |  |  |  | P [35] | *Caretta caretta* | Florida (USA) |
| *Amphiorchis* sp. |  | KX987107 |  |  |  |  |  |  |  | P [36] | *Caretta caretta* | Valencia (Spain) |
| *Amphiorchis* sp. |  | KX987109 |  |  |  |  |  |  |  | P [36] | *Caretta caretta* | Florida (USA) |
| *Amphiorchis* sp. |  | KX987111 |  |  |  |  |  |  |  | P [36] | *Thylaeodus cf. rugulosus* | Valencia (Spain) |
| *Amphiorchis* sp. |  |  |  |  |  | KX987108 |  |  |  | P [36] | *Caretta caretta* | Valencia (Spain) |
| *Amphiorchis* sp. |  |  |  |  |  | KX987110 |  |  |  | P [36] | *Caretta caretta* | Florida (USA) |
| *Amphiorchis* sp. |  |  |  |  |  | KX987112 |  |  |  | P [36] | *Thylaeodus cf. rugulosus* | Valencia (Spain) |
| *Baracktrema obamai* Roberts, Platt & Bullard, 2016 |  | KX061500 | KY243883 |  |  |  |  |  |  | P [37], P [1] | *Siebenrockiella crassicollis* | Malaysia |
| *Carettacola hawaiiensis* Dailey, Fast & Balazs, 1991 |  | KU600068 | AY604709 |  |  | KU600075 |  |  |  | P [35], P [38] | *Chelonia mydas* | Queensland (Australia)/Pacific Ocean Hawaii (USA) |
| **Taxon** |  | **28S** | | |  | **ITS2** | | |  | **Source** | **Host** | **Country** |
| *Carettacola* sp. |  | KU600070 |  |  |  |  |  |  |  | P [35] | *Chelonia mydas* | Queensland (Australia) |
| *Carettacola* sp. |  | KU600069 |  |  |  |  |  |  |  | P [35] | *Chelonia mydas* | Queensland (Australia) |
| *Carettacola* sp. |  |  |  |  |  | KU600076 |  |  |  | P [35] | *Chelonia mydas* | Queensland (Australia) |
| *Carettacola* sp. |  |  |  |  |  | KU600077 |  |  |  | P [35] | *Chelonia mydas* | Queensland (Australia) |
| *Coeuritrema platti* Roberts, Orélis-Ribeiro, Dang, Halanych & Bullard, 2016 |  | KX712243 |  |  |  |  |  |  |  | P [39] | *Pelodiscus sinensis* | Vietnam |
| *Hapalorhynchus foliorchis* Brooks & Mayes, 1975 |  | KX712242 |  |  |  |  |  |  |  | P [39] | *Chelydra serpentina* | Auburn, Alabama (USA) |
| *Hapalorhynchus gracilis* Stunkard, 1922 |  | AY604710 |  |  |  |  |  |  |  | P [38] | *Chelydra serpentina* | Reelfoot Lake, Tennessee (USA) |
| *Platydidymus flecterotestis* (Zhukov, 1971) |  | HE806362 |  |  |  |  |  |  |  | U [40] | n.d. | Kievka River (Russia) |
| *Hapalotrema mehrai* Rao, 1976; junior synonym of *H. pambanensis* (see Chapman et al., 2015) |  | AY604708 |  |  |  |  |  |  |  | P [38] | *Chelonia mydas* | Pacific Ocean Hawaii (USA) |
| *Hapalotrema mistroides* (Monticelli, 1896) |  | KU892016 |  |  |  | GU937893 | KY499798 |  |  | P [35], P [41], [42] | *Caretta caretta* | Florida (USA)/Latium (Italy) |
| ***Hapalotema* *mistroides* (Monticelli, 1896)** **Italy** |  | **LT882715** |  |  |  | **LT617052** |  |  |  | **P [43]** | *Caretta caretta* | Northern Adriatic Coast (Italy) |
| *Hapalotrema pambanensis* Mehrotra, 1973 |  | KM652620 |  |  |  | KM652626 | KM652616 |  |  | P [44] |  | Queensland (Australia)/ Hawaii (USA) |
| *Hapalotrema postorchis* Rao, 1976; |  | KM652621 |  |  |  | GU937895 | KM652617 |  |  | P [41], P [44] | *Chelonia mydas* | Queensland (Australia)/USA |
| *Hapalotrema synorchis* Luhman, 1935 |  | KM652622 |  |  |  | KM652618 | GU937894 | KT361641 |  | P [44], P [41],U [45] | *n.d./Caretta caretta/Eretmochelys imbricata* | Queensland (Australia)/USA/Northern territory (Australia) |
| *Learedius learedi* Price, 1934 |  | KM652625 | KM652623 | AY604707 |  | KM652619 | GU937892 | KM652627 |  | P [44], P [38], P [41] | *n.d./Chelonia mydas/n.d.* | Hawaii (USA)/ Queensland (Australia)/USA |
| *Neospirorchis* sp. |  | KU600072 |  |  |  |  |  |  |  | P [35] | *Chelonia mydas* | Queensland (Australia) |
| *Neospirorchis* sp. |  |  |  |  |  | KU600079 |  |  |  | P [35] | *Chelonia mydas* | Queensland (Australia) |
| *Neospirorchis* sp. Neospirgen2 |  | KU600073 |  |  |  | KU600080 |  |  |  | P [35] | *Chelonia mydas* | Queensland (Australia) |
| *Neospirorchis* sp. Neospirgen3 |  | KU600074 |  |  |  | KU600081 |  |  |  | P [35] | *Chelonia mydas* | Queensland (Australia) |
| ***Neospirorchis* sp. Italy** |  | **LT882716** |  |  |  | **LT617053** |  |  |  | **P [43]** | *Caretta caretta* | Northern Adriatic Coast (Italy) |
| *Neospirorchis* sp. Neogen1 |  |  |  |  |  | KU601323 |  |  |  | U [46] | *Caretta caretta/Lepidochelys kempii* | Florida (USA) |
| *Neospirorchis* sp. Neogen2 |  |  |  |  |  | KU601324 |  |  |  | U [46] | *Caretta caretta* | Florida (USA) |
| *Neospirorchis* sp. Neogen3 |  |  |  |  |  | KU601325 |  |  |  | U [46] | *Chelonia mydas* | Florida (USA) |
| *Neospirorchis* sp. Neogen3var |  |  |  |  |  | KU601326 |  |  |  | U [46] | *Chelonia mydas* | Florida (USA) |
| *Neospirorchis* sp. Neogen4 |  |  |  |  |  | KU601327 |  |  |  | U [46] | *Caretta caretta* | Florida (USA) |
| *Neospirorchis* sp. Neogen5 |  |  |  |  |  | KU601328 |  |  |  | U [46] | *Caretta caretta/Lepidochelys kempii* | Florida (USA) |
| *Neospirorchis* sp. Neogen6 |  |  |  |  |  | KU601329 |  |  |  | U [46] | *Caretta caretta* | Florida (USA) |
| *Neospirorchis* sp. Neogen6var |  |  |  |  |  | KU601330 |  |  |  | U [46] | *Caretta caretta* | Florida (USA) |
| *Neospirorchis* sp. Neogen7 |  |  |  |  |  | KU601331 |  |  |  | U [46] | *Chelonia mydas* | Florida (USA) |
| *Neospirorchis* sp. Neogen8 |  |  |  |  |  | KU601332 |  |  |  | U [46] | *Caretta caretta* | Florida (USA) |
| *Neospirorchis* sp. Neogen9 |  |  |  |  |  | KU601333 |  |  |  | U [46] | *Caretta caretta/Lepidochelys kempii* | Florida (USA) |
| *Neospirorchis* sp. Neogen10 |  |  |  |  |  | KU601334 |  |  |  | U [46] | *Caretta caretta* | Florida (USA) |
| *Neospirorchis* sp. Neogen11 |  |  |  |  |  | KU601335 |  |  |  | U [46] | *Caretta caretta* | Florida (USA) |
| *Neospirorchis* sp. Neogen12 |  |  |  |  |  | KU601336 |  |  |  | U [46] | *Caretta caretta* | Florida (USA) |
| *Neospirorchis* sp. Neogen13 |  |  |  |  |  | KU601337 |  |  |  | U [46] | *Caretta caretta/Chelonia mydas* | Florida (USA) |
| *Neospirorchis* sp. Neogen14 |  |  |  |  |  | KU601338 |  |  |  | U [46] | *Chelonia mydas* | Florida (USA) |
| *Neospirorchis* sp. Neogen15 |  |  |  |  |  | KU601339 |  |  |  | U [46] | *Caretta caretta* | Florida (USA) |
| *Neospirorchis* sp. Neogen16 |  |  |  |  |  | KU601340 |  |  |  | U [46] | *Chelonia mydas* | Florida (USA) |
| *Neospirorchis* sp. Neogen17 |  |  |  |  |  | KU601341 |  |  |  | U [46] | *Chelonia mydas* | Florida (USA) |
| *Neospirorchis* sp. Neogen19 |  |  |  |  |  | KU601343 |  |  |  | U [46] | *Chelonia mydas* | Florida (USA) |
| *Neospirorchis* sp. Neogen20 |  |  |  |  |  | KU601344 |  |  |  | U [46] | *Eretmochelys imbricata* | Florida (USA) |
| *Neospirorchis* sp. Neogen18 |  |  |  |  |  | KU601342 |  |  |  | U [46] | *Chelonia mydas* | Florida (USA) |
| *Spirhapalum polesianum* Ejsmont, 1927 |  | AY604705 |  |  |  |  |  |  |  | P [38] | *Emys orbicularis* | Lesniki, Kyivi Region (Ukraine) |
| *Spirhapalum siamensis* Tkach, Snyder & Vaughan, 2009 |  | FJ481166 |  |  |  |  |  |  |  | P [47] | *Cuora amboinensis* | Thailand |
| Spirorchiidae sp. |  | GU270101 |  |  |  |  |  |  |  | U [48] | n.d. | Bosque del Apache, New Mexico (USA) |
| Spirorchiidae sp. |  | FJ550134 |  |  |  |  |  |  |  | U [49] | *Physa acuta* | USA |
| Spirorchiidae sp. |  | KU600071 |  |  |  |  |  |  |  | P [35] | *Chelonia mydas* | Queensland (Australia) |
| Spirorchiidae sp. |  |  |  |  |  | KU600078 |  |  |  | P [35] | *Chelonia mydas* | Queensland (Australia) |
| Spirorchid sp. |  | AY858884 |  |  |  |  |  |  |  | P [50] | n.d. | n.d. |
| *Spirorchis artericola* (Ward, 1921) |  | AY604704 |  |  |  |  |  |  |  | P [38] | *Chrysemys picta* | Reefoot Lake, Tennessee (USA) |
| *Spirorchis collinsi* Roberts & Bullard, 2016 |  | KY091664 |  |  |  |  |  |  |  | P [51] | *Deirochelys reticularia* | Tuskegee, Alabama (USA) |
| *Spirorchis haematobium* (Stunkard, 1922) |  | FJ481164 |  |  |  |  |  |  |  | P [47] | *Chelydra serpentina* | USA |
| *Spirorchis picta* Stunkard, 1923 |  | KY091665 |  |  |  |  |  |  |  | P [51] | *Trachemys scripta* | Auburn, Alabama (USA) |
| *Spirorchis* cf. *scripta* |  | KY091666 |  |  |  |  |  |  |  | P [51] | *Deirochelys reticularia* | Tuskegee, Alabama (USA) |
| *Spirorchis scripta* Stunkard, 1923 |  | AY858882 | AY222174 |  |  |  |  |  |  | P [50], P [52] | *Trachemys scripta scripta* | USA |
| *Spirorchis* sp. |  | KM507474 |  |  |  | KM507474 |  |  |  | P [53] | *Terrapene carolina* | Florida (USA) |
| *Unicaecum* sp. |  | AY604711 |  |  |  |  |  |  |  | P [38] | *Trachemys scripta* | Reefoot Lake, Tennessee (USA) |
| *Vasotrema robustum* Stunkard, 1928 |  | AY604706 | AY858883 |  |  |  |  |  |  | P [38], P [50] | *Apalone spinifera* | Nishnabotna River, Iowa (USA) |

P, published reference; U, unpublished; n.d., not determined.

**References**

[1] Orelis-Ribeiro R, Halanych KM, Dang BT, Bakenhaster MD, Arias CR, Bullard SA. Two new species of *Elopicola* (Digenea: Aporocotylidae) from Hawaiian ladyfish, *Elops* *hawaiensis* (Eastern Sea) and Atlantic tarpon, *Megalops atlanticus* (Gulf of Mexico) with a comment on monophyly of elopomorph blood flukes,. Parasitol. Int. 2017;66(3):305-318.

[2] Holzer AS, Montero FE, Repulles A, Nolan MJ, Sitja-Bobadilla A, Alvarez-Pellitero P, Zarza C, Raga JA. *Cardicola aurata* sp. n. (Digenea: Sanguinicolidae) from Mediterranean *Sparus aurata* L. (Teleostei: Sparidae) and its unexpected phylogenetic relationship with *Paradeontacylix* McIntosh, 1934. Parasitol. Int. 2008;57 (4), 472-482.

[3] Yong RQ, Cutmore SC, Miller TL, Wee NQ, Cribb TH. A complex of *Cardicola* Short, 1953 (Digenea: Aporocotylidae) species infecting the milkfish *Chanos chanos* Forsskal (Gonorynchiformes), with descriptions of two new species. Syst. Parasitol. 2016;93 (9), 831-846.

[4] Shiraksahi S, Tani K, Ishimaru K, Shin SP, Honryo T, Uchida H, Ogawa K. Discovery of intermediate hosts for two species of blood flukes *Cardicola orientalis* and *Cardicola* *forsteri* (Trematoda: Aporocotylidae) infecting Pacific bluefin tuna in Japan. [Parasitol Int.](https://www.ncbi.nlm.nih.gov/pubmed/?term=The+discovery+of+the+intermediate+hosts+of+Cardicola+orientalis+and+Cardicola+forsteri+%28Trematoda%3A+Aporocotylidae%29%2C+blood+flukes+infecting+Pacific+bluefin+tuna+in+Japan.) 2016;(2):128-36.

[5] Palacios-Abella JF, Rodriguez-Llanos J, Mele S, Montero FE. Morphological characterisation and identification of four species of *Cardicola* Short, 1953 (Trematoda: Aporocotylidae) infecting the Atlantic bluefin tuna *Thunnus thynnus* (L.) in the Mediterranean Sea. Syst. Parasitol. 2015;91 (2), 101-117.

[6] Nolan MJ, Cribb TH. *Cardicola* Short, 1953 and *Braya* n. gen. (Digenea: Sanguinicolidae) from Five Families of Tropical Indo-Pacific Fishes. Zootaxa 2006;1265, 3-80.

[7] Repulles-Albelda A, Montero FE, Holzer AS, Ogawa K, Hutson KS, Raga JA. Speciation of the *Paradeontacylix* spp. (Sanguinicolidae) of *Seriola dumerili*. Two new species of the genus *Paradeontacylix* from the Mediterranean. Parasitol. Int. 2008;57 (3), 405-414.

[8] Warren MB, Orelis-Ribeiro R, Ruiz CF, Dang T, Arias CR, Bullard SA. Endocarditis associated with blood fluke infections (Digenea: Aporocotylidae: *Psettarium* cf. *anthicum*) among aquacultured cobia (*Rachycentron canadum*) from Nha Trang Bay, Vietnam. Aquaculture 2017;468, 549-557.

[9] Nolan MJ, Cantacessi C, Cutmore SC, Cribb TH, Miller TL. High-intensity cardiac infections of *Phthinomita heinigerae* n. sp. (Digenea: Aporocotylidae) in the orangelined cardinalfish, *Taeniamia fucata* (Cantor), off Heron Island on the Great Barrier Reef. Parasitol. Int. 2016;65(5 Pt A):371-7.

[10] Nolan MJ, Cribb TH. An exceptionally rich complex of Sanguinicolidae Von Graff, 1907 (Platyhelminthes: Trematoda) from Siganidae, Labridae and Mullidae (Teleostei: Perciformes) from the Indo-West Pacific Region. Unpublished.

[11] Yong RQ, Cutmore SC, Bray RA, Miller TL, Semarariana IW, Palm HW, Cribb TH. Three new species of blood flukes (Digenea: Aporocotylidae) infecting pufferfishes (Teleostei: Tetraodontidae) from off Bali, Indonesia. Parasitol. Int. 2016;65(5 Pt A):432-43.

[12] Hall KA, Ogawa K. Unpublished.

[13] Brant SV. The occurrence of the avian schistosome *Allobilharzia visceralis* Kolarova, Rudolfova, Hampl et Skirnisson, 2006 (Schistosomatidae) in the tundra swan, *Cygnus columianus* (Anatidae) from North America. [Folia Parasitol (Praha)](https://www.ncbi.nlm.nih.gov/pubmed/17886738) 2007;54(2):99-104.

[14] Brant SV, Jouet D, Ferte H, Loker ES. *Anserobilharzia* gen. n. (Digenea, Schistosomatidae) and redescription of *A. brantae* (Farr & Blankemeyer, 1956) comb. n. (syn. *Trichobilharzia brantae*), a parasite of geese (Anseriformes). [Zootaxa.](https://www.ncbi.nlm.nih.gov/pubmed/?term=Anserobilharzia+gen.+n.+%28Digenea%2C+Schistosomatidae%29+and+redescription+of+A.+brantae) 2013;3670:193-206.

[15] Al-Kandari WY, Al-Bustan SA, Isaac AM, George BA, Chandy BS. Molecular identification of *Austrobilharzia* species parasitizing *Cerithidea cingulata* (Gastropoda: Potamididae) from Kuwait Bay. J. Helminthol. 2012;86(4):470-8.

[16] Brant SV, Pomajbikova K, Modry D, Petrzelkova KJ, Todd A, Loker ES. Molecular phylogenetics of the elephant schistosome *Bivitellobilharzia loxodontae* (Trematoda: Schistosomatidae) from the Central African Republic. J. Helminthol. 2013;87(1):102-7. 2012.

[17] Devkota R, Brant SV, Thapa A, Loker ES. Sharing schistosomes: the elephant schistosome *Bivitellobilharzia nairi* also infects the greater one-horned rhinoceros (*Rhinoceros unicornis*) in Chitwan National Park, Nepal. J. Helminthol. 2014;88(1):32-40.

[18] Brant SV, Bochte CA, Loke ES. New Intermediate Host Records for the Avian Schistosomes *Dendritobilharzia pulverulenta*, *Gigantobilharzia huronensis*, and *Trichobilharzia querquedulae* from North America. J. Parasitol. 2011;97 (5), 946-949.

[19] Rudolfova J, Hampl V, Bayssade-Dufour C, Lockyer A.E, Littlewood DT, Horak P. Validity reassessment of *Trichobilharzia* species using *Lymnaea stagnalis* as the intermediate host. Parasitol. Res. 2005;95 (2), 79-89.

[20] Schuster RK, Aldhoun JA, O'Donovan D. *Gigantobilharzia melanoidis* n.sp. (Trematoda: Schistosomatidae) from *Melanoides* *tuberculata* (Gastropoda: Thiaridae) in the United Arab Emirates. Parasitol. Res. 2014;113 (3), 959-972.

[21] Littlewood DT, Johnston DA. Molecular phylogenetics of the four *Schistosoma* species groups determined with partial 28S ribosomal RNA gene sequences. Parasitology 1995;111 (PT 2), 167-175.

[22] Wang CR, Li L, Ni HB, Zhai YQ, Chen AH, Chen J, Zhu XQ. *Orientobilharzia turkestanicum* is a member of *Schistosoma* genus based on phylogenetic analysis using ribosomal DNA sequences. Exp. Parasitol. 2009;121 (2), 193-197.

[23] Hanelt B, Brant SV, Steinauer ML, Maina GM, Kinuthia JM, Agola LE, Mwangi IN, Mungai BN, Mutuku MW, Mkoji GM, Loker ES. *Schistosoma kisumuensis* n. sp. (Digenea: Schistosomatidae) from murid rodents in the Lake Victoria Basin, Kenya and its phylogenetic position within the *S. haematobium* species group. Parasitology 2009;136 (9), 987-1001.

[24] Barber KE, Mkoji GM, Loker ES. PCR-RFLP analysis of the ITS2 region to identify *Schistosoma haematobium* and *S. bovis* from Kenya. [Am J Trop Med Hyg.](https://www.ncbi.nlm.nih.gov/pubmed/?term=RFLP+analysis+of+the+ITS2+region+to+identify+Schistosoma+haematobium+and+S.+bovis+from+Kenya) 2000;62(4):434-40.

[25] Bowles J, Blair D, McManus DP. A molecular phylogeny of the human schistosomes. Mol. Phylogenet. Evol. 1995;4 (2), 103-109.

[26] Morgan JA, DeJong RJ, Kazibwe F, Mkoji GM, Loker ES. A newly-identified lineage of *Schistosoma*. Int. J. Parasitol. 2003;33 (9), 977-985.

[27] Webster BL, Littlewood DT. Mitochondrial gene order change in *Schistosoma* (Platyhelminthes: Digenea: Schistosomatidae). Int. J. Parasitol. 2012;42 (3), 313-321.

[28] Zhao QP, Jiang MS, Dong HF, Nie P. Diversification of *Schistosoma japonicum* in Mainland China Revealed by Mitochondrial DNA. PLoS Negl Trop Dis 2012;6 (2), E1503.

[29] Webster BL, Webster JP, Gouvras AN, Garba A, Lamine MS, Diaw OT, et al. DNA 'barcoding' of *Schistosoma mansoni* across sub-Saharan Africa supports substantial within locality diversity and geographical separation of genotypes. Acta Trop. 2013;128 (2), 250-260.

[30] Devkota R, Brant SV, Loker ES. The *Schistosoma indicum* species group in Nepal: presence of a new lineage of schistosome and use of the *Indoplanorbis exustus* species complex of snail hosts. Int. J. Parasitol. 2015;45(13):857-70.

[31] Morgan JA, DeJong RJ, Lwambo NJ, Mungai BN, Mkoji GM, Loker ES. First report of a natural hybrid between *Schistosoma mansoni* and *S. rodhaini*. J. Parasitol. 2003;89 (2), 416-418.

[32] Lawton SP, Lim RM, Dukes JP, Cook RT, Walker AJ, Kirk RS. Identification of a major causative agent of human cercarial dermatitis, *Trichobilharzia franki* (Muller and Kimmig 1994), in southern England and its evolutionary relationships with other European populations. Parasit Vectors 2014;7 (1), 277.

[33] Brant SV, Loker ES. Molecular systematics of the avian schistosome genus *Trichobilharzia* (Trematoda: Schistosomatidae) in North America. J. Parasitol. 2009;95 (4), 941-963.

[34] Pinto HA, Brant SV, de Melo AL. *Physa marmorata* (Mollusca: Physidae) as a natural intermediate host of *Trichobilharzia* (Trematoda: Schistosomatidae), a potential causative agent of avian cercarial dermatitis in Brazil. Acta Trop. 2014;138, 38-43.

[35] Chapman PA, Traub RJ, Kyaw-Tanner MT, Owen H, Flint M, Cribb TH, Mills PC. Terminal Restriction Fragment Length Polymorphism for the Identification of Spirorchiid Ova in Tissues from the Green Sea Turtle, *Chelonia mydas*. PLoS ONE 2016;11 (8), E0162114.

[36] Cribb TH, Crespo-Picazo JL, Cutmore SC, Stacy BA, Chapman PA, Garcia-Parraga D. Elucidation of the first definitively identified life cycle for a marine turtle blood fluke (Trematoda: Spirorchiidae) enables informed control. Int. J. Parasitol. 2017;47(1):61-67.

[37] Roberts JR, Platt TR, Bullard SA. New genus of blood fluke (Digenea: Schistosomatoidea) from Malaysian freshwater turtle (Geoemydidae) and its phylogenetic position within Schistosomatoidea. J. Parasitol. 2016;102(4):451-62.

[38] Snyder SD. Phylogeny and paraphyly among tetrapod blood flukes (Digenea: Schistosomatidae and Spirorchiidae). Int. J. Parasitol. 2004;34 (12), 1385-1392.

[39] Roberts JR., Orelis-Ribeiro R, Dang BT., Halanych KM, Bullard SA. Blood flukes of Asiatic softshell turtles: revision of *Coeuritrema* Mehra, 1933 (Digenea: Schistosomatoidea) and a new species infecting Chinese softshell turtles, *Pelodiscus sinensis* (Trionychidae), from Vietnam. Folia Parasitol. 2016;63.

[40] Besprozvannykh VV, Ermolenko AV, Atopkin DM. Redescription of trematodes *Skrjabinolecithum spasskii* Belouss, 1954, *S. flecterotestis* (Zhukov, 1971) comb. nov. (Trematoda:. Haploporidae Nicoll, 1914) and new species *Skrjabinolecithum* sp. from mullet of South of the Russian Far East. Unpublished.

[41] Stacy BA, Frankovich T, Greiner E, Alleman,AR, Herbst LH,Klein P, Bolten A, McIntosh A, Jacobson ER. Detection of Spirorchiid trematodes in gastropod tissues by polymerase chain reaction: preliminary identification of an intermediate host of *Learedius learedi*. [J Parasitol.](https://www.ncbi.nlm.nih.gov/pubmed/?term=Detection+of+Spirorchiid+Trematodes+in+Gastropod+Tissues+by) 2010;96(4):752-7.

[42] Santoro M, Di Nocera F, Iaccarino D, Lawton SP, Cerrone A, Degli Uberti B, D'Amore M, Affuso A, Hochscheid S, Maffucci F, Galiero G. Pathology and molecular analysis of *Hapalotrema mistroides* (Digenea: Spirorchiidae) infecting a Mediterranean loggerhead turtle *Caretta caretta*. Dis. Aquat. Org. 2017;124 (2), 101-108.

[43] Marchiori et al., this paper

[44] Chapman PA, Cribb TH, Blair D, Traub RJ, Kyaw-Tanner MT, Flint M, Mills PC. Molecular analysis of the genera *Hapalotrema* Looss, 1899 and *Learedius* Price, 1934 (Digenea: Spirorchiidae) reveals potential cryptic species, with comments on the validity of the genus *Learedius*. Syst. Parasitol. 2015;90 (1), 67-79.

[45] Barton DP, Chapman PA, Melville L, Groom RA. Report of the presence of *Hapalotrema synorchis* (Digenea:Spirorchiidae) in the hawksbill turtle *Eretmochelys imbricata* (Reptilia: Cheloniidae) in Northern Territory waters. Unpublished

[46] Stacy BA, Chapman PA, Foley AM, Greiner EC, Herbst LH, Bolten AB, Klein PA, Manire CA, Jacobson ER. Genetic diversity of the genus *Neospirorchis* (Spirorchiidae) in sea turtles and correlation with anatomic location and definitive host species. Unpublished

[47] Tkach VV, Snyder SD, Vaughan JA. A new species of blood fluke (Digenea: Spirorchiidae) from the Malayan box turtle, *Cuora amboinensis*, (Cryptodira: Geomydidae) in Thailand. J Parasitol. 2009;95(3):743-6.

[48] Goodman KJ, Lovato TL, Montano ET, Zalar RL, Morriss GR,Natvig DO, Brant SV, Adema CM. Unpublished.

[49] Kraus TJ, Brant SV, Adema CM. Trematode cercariae from physid snails from the Middle Rio Grande. Unpublished.

[50] Brant SV, Morgan JA, Mkoji GM, Snyder SD, Rajapakse RP, Loker ES. An approach to revealing blood fluke life cycles, taxonomy, and diversity: provision of key reference data including DNA sequence from single life cycle stages. J. Parasitol. 2006;92 (1), 77-88.

[51] Roberts JR, Orelis-Ribeiro R, Halanych KM, Arias CR, Bullard SA. A new species of *Spirorchis* MacCallum, 1918 (Digenea: Schistosomatoidea) and *Spirorchis* cf. *scripta* from chicken turtle, *Deirochelys reticularia* (Emydidae), with an emendation and molecular phylogeny of *Spirorchis*. Folia Parasitol. 2016;63, 041.

[52] Olson PD, Cribb TH, Tkach VV, Bray RA, Littlewood DT. Phylogeny and classification of the Digenea (Platyhelminthes: Trematoda). Int. J. Parasitol. 2003;33 (7), 733-755.

[53] Yonkers SB, Schneider R, Reavill DR, Archer LL, Childress AL, Wellehan JFX Jr. Coinfection with a novel fibropapilloma-associated herpesvirus and a novel *Spirorchis* sp. in an eastern box turtle (*Terrapene carolina*) in Florida. [J Vet Diagn Invest.](https://www.ncbi.nlm.nih.gov/pubmed/?term=Coinfection+with+a+Novel+Fibropapilloma-Associated+Herpesvirus) 2015 Jul;27(4):408-13.
